# Effect of Fluorescence Lymph Node Mapping on Improving Diagnostic Values of CT D3 Lymph Node Staging for Right-Sided Colon Cancer

**DOI:** 10.3390/cancers16203496

**Published:** 2024-10-16

**Authors:** Gyung Mo Son, Tae Un Kim, Mi Sook Yun, ChangYeop Kim, In Young Lee, Su Bum Park, Dong-Hoon Shin, Gi Won Ha

**Affiliations:** 1 Department of Surgery, Pusan National University Yangsan Hospital, Pusan National University School of Medicine, Yangsan 50612, Republic of Korea; babe123415@gmail.com; 2 Research Institute for Convergence of Biomedical Science and Technology, Pusan National University Yangsan Hospital, Yangsan 50612, Republic of Korea; msyun@pusan.ac.kr (M.S.Y.); vmffkdl38@naver.com (I.Y.L.); 3 Department of Radiology, Pusan National University Yangsan Hospital, Pusan National University School of Medicine, Yangsan 50612, Republic of Korea; kimtaeun78@hanmail.net; 4 Department of Internal Medicine, Pusan National University Yangsan Hospital, Pusan National University School of Medicine, Yangsan 50612, Republic of Korea; psubumi@hanmail.net; 5 Department of Pathology, Pusan National University Yangsan Hospital, Pusan National University School of Medicine, Yangsan 50612, Republic of Korea; donghshin@pusan.ac.kr; 6 Research Institute of Clinical Medicine of Jeonbuk National University-Biomedical Research Institute of Jeonbuk National University Hospital, Jeonju 54907, Republic of Korea; acts29@jbnu.ac.kr

**Keywords:** fluorescence, lymph nodes, indocyanine green, colonic neoplasms, computed tomography, neoplasm staging

## Abstract

Background/Objectives: This study evaluated the impact of fluorescence lymph node mapping (FLNM) using indocyanine green (ICG) on the diagnostic accuracy of preoperative computed tomography (CT) in right-sided colon cancer. Methods: A total of 218 patients who underwent laparoscopic right hemicolectomy with D3 lymph node dissection (LND) were analyzed: 86 patients in the FLNM group and 132 in the conventional surgery group. The FLNM technique allowed for enhanced intraoperative visualization of lymph node (LN) and more precise dissection, improving the identification of metastatic LNs. The diagnostic value of preoperative CT staging was assessed in both the FLNM and control groups by calculating the apparent prevalence, true prevalence, sensitivity, specificity, positive predictive value (PPV), negative predictive value, positive likelihood ratio (PLR), negative likelihood ratio, false positive and false negative proportions, and accuracy. Results: FLNM increased the accuracy of CT staging for detecting D3 LN metastasis in advanced cancer cases, with a higher PPV, PLR, and accuracy. In the FLNM group, the false-positive rate was significantly reduced, and the specificity was higher compared to the control group. Multivariate analysis identified FLNM as an independent factor associated with improved D3 LN metastasis detection. These findings suggest that incorporating FLNM into surgical procedures enhances the diagnostic value of preoperative CT by improving the precision of LND, particularly in patients with advanced colon cancer. Conclusions: The use of FLNM for D3 LND enhances the diagnostic accuracy of cN staging in right-sided colon cancer by improving surgical precision.

## Simple Summary

Fluorescence lymph node mapping using indocyanine green significantly enhances the accuracy of preoperative CT staging in right-sided colon cancer by providing a real-time visualization of lymph nodes, which allows for more precise identification and removal of potentially metastatic nodes, particularly in advanced cases; this approach reduces false positives, decreases over-staging, and improves the alignment between CT findings and pathologic outcomes, thereby leading to better diagnostic accuracy and more effective treatment planning.

## 1. Introduction

Considering right-sided colon cancer is one of the most common forms of colorectal cancer, the frequency of right hemicolectomies (RHC) has been increasing [1]. In cases of advanced colon cancer, D3 lymph node dissection (LND), which involves dissection of the lymph nodes (LN) around the origin of the colon’s main feeding vessels, typically from the superior mesenteric artery (SMA), is the standard oncological treatment [2]. It involves central vascular ligation and dissection around the SMA and superior mesenteric vein (SMV). However, this procedure carries a high risk of vascular injury and bleeding, particularly for less experienced surgeons [3]. Furthermore, the necessity of dissecting the lymphatic tissue around the SMV and SMA remains debatable [4].

Abdominal computed tomography (CT) is essential for determining the extent of LND in locally advanced colon cancer, particularly in clinical T3, N1, or higher statuses, for which a D3 LND is recommended [5,6,7]. While the CT accuracy for clinical T3/4 staging exceeds 80–90%, its accuracy for clinical N (cN) staging remains low at 60–70% [8,9,10]. The characteristics of metastatic LNs, such as size, shape, and matrix heterogeneity, assist in radiological cN staging [11]. However, reactive lymphadenopathy or small metastatic foci can result in false positives or negatives [12]. Traditionally, the limited accuracy of cN staging relative to pathologic N (pN) staging has overlooked the quality of LND, which serves as a bridge between radiological and pathologic staging. Adequate LND enhances the detection of metastatic LNs and potentially improves adjuvant chemotherapy decisions and survival in patients with advanced colon cancer [13].

Fluorescence lymph node mapping (FLNM) using indocyanine green (ICG) has emerged as a promising technique for enhancing the visualization of LNs connected to primary tumors during surgery [14]. FLNM highlights the lymphatic flow pathway to the D3 LNs, aiding in defining the extent of D3 LND and enabling a more tailored approach to D3 LND in each patient [15]. FLNM can significantly increase the harvested D3 LN count without increasing the risk of vascular injury. In addition, FLNM demonstrates an improved detection rate of metastatic D3 LNs [16].

We hypothesized that FLNM improves the diagnostic accuracy of cN staging by improving the precision of D3 LND. Therefore, this study aimed to evaluate the effect of FLNM-guided D3 LND on the diagnostic value of cN staging in right-sided colon cancer.

## 2. Materials and Methods

### 2.1. Patients

**Figure 1:**
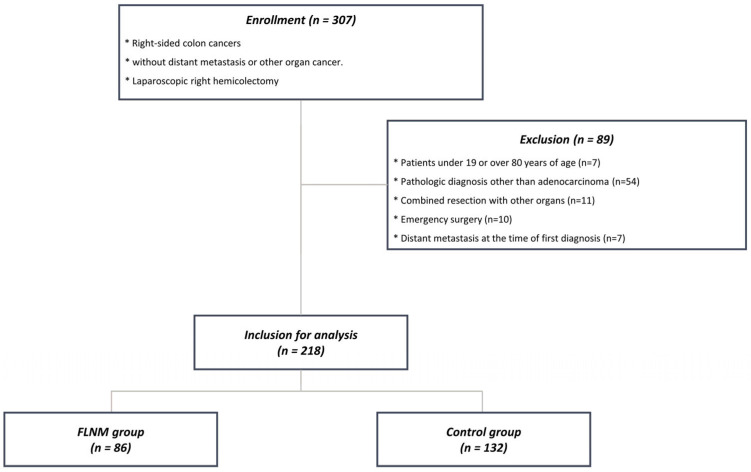
Patient flowchart illustrating the enrollment and allocation of participants in the study.

We enrolled 307 patients who underwent laparoscopic RHC between January 2018 and December 2023 at the Pusan National University Yangsan Hospital, Korea. The inclusion criteria were patients aged 19–80 years with right-sided colon cancer and no distant metastasis or other organ cancers. Eighty-nine patients were excluded based on exclusion criteria. Of the 218 included patients, 86 underwent FLNM and 132 underwent conventional radical surgery without FLNM (Figure 1). Right-sided colon cancer was defined as a tumor located in the cecum, ascending colon, hepatic flexure, or proximal transverse colon. This study was approved by the Institutional Review Board of Pusan National University Yangsan Hospital (IRB No. 05-2023-163) and written informed consent was obtained from all the patients.

### 2.2. Preoperative Abdominal CT Staging

A radiologist was assigned the task of determining the “T” and “N” stages of the tumor, in accordance with the staging system established by the World Health Organization (WHO). The WHO guidelines classify tumor staging with the indicator “T”. cT_1-2_ tumors are characterized by intraluminal masses without evidence of extraluminal extension or bowel wall deformation. cT_3_ tumors are identified by their smooth or nodular extension beyond the normal contours of the bowel wall but without a spiculated appearance. cT_4_ tumors are defined as tumors that extend into the adjacent peritoneum or infiltrate nearby tissues or organs. An LN was classified as metastatic and included in the count if it displayed the following characteristics: internal heterogeneity, an irregular outer border, a rounded shape, and the presence of a cluster of three or more LNs. Size was not used as a criterion because of the unclear reference points and low accuracy. Furthermore, the small diameter of the LN on D3 region was considered for metastatic LN staging [17]. CT staging was performed by a radiologist with substantial experience in colon cancer staging, who was blinded to the pathologic staging results.

### 2.3. Fluorescence Lymph Nodes Mapping

**Figure 2:**
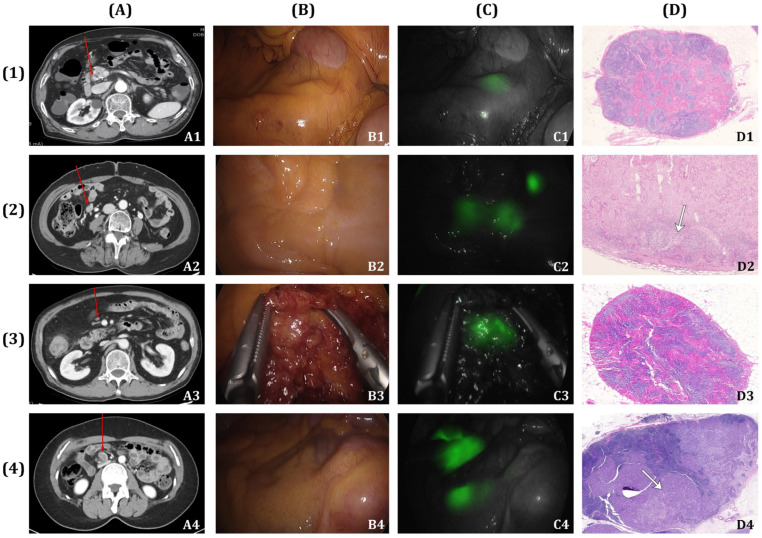
Images of D3 lymph node (LN) with and without metastasis. (**A**) CT image of a D3 LN. (**B**) D3 LN under white light during surgery. (**C**) ICG fluorescence image of the D3 LN obtained with a laparoscopic near-infrared camera. (**D**) Pathologic image of the D3 LN (H&E staining). (1) Patient with no D3 LN metastasis on both CT and pathologic images. (2) Patient with no metastasis on CT but with metastasis on the pathologic image. (3) Patient with suspicious D3 LN metastasis on CT but no metastasis on the pathologic image. (4) Patient with D3 LN metastasis on both CT and pathologic images. The red arrow indicates the LNs observed in the D3 region on CT imaging. White arrows indicate areas within the LNs containing cancer cells. Cases (2) and (3) illustrate discrepancies between CT and pathologic images in detecting D3 LN metastasis.

For FLNM, an endoscopic submucosal injection of ICG (DIAGNOGREEN INJ. 25 mg; Daiichi Sankyo, Tokyo, Japan) was administered at two sites near the distal margin of the colon cancer, 12–18 h before surgery. The ICG solution was diluted to 0.25 mg/mL, with a total volume of 1–2 mL, based on a previous study [16,18]. During surgery, ICG fluorescence was visualized using a laparoscopic near-infrared (NIR) camera (1588 AIM camera system; Stryker, Kalamazoo, MI, USA). The path of the ICG is as follows: ICG drains into the regional LNs (D1), passes through the intermediate LNs (D2), and reaches the apical D3 LNs at the roots of the ileocolic artery (ICA), right colic artery (RCA), and middle colic artery (MCA) [13]. The ICG-stained areas of the D3 LNs defines the extent of D3 LND (Figure 2).

All the patients underwent laparoscopic RHC with D3 LND. In the FLNM group, fluorescent lymphatic drainage pathways and D3 LN distribution were explored before dissection. The medial extent of D3 LND was determined by the location of the fluorescent LNs, ensuring the removal of all fluorescent nodes at the ICA, RCA, and MCA roots. If fluorescent nodes were found behind the MCA root of the SMA, the deep lymphatic tissue was removed [19]. After dissection, the absence of residual fluorescent LNs confirmed the completeness of LN dissection. In the control group, the medial extent of D3 LND was determined using anatomical landmarks, with the medial boundary set along an imaginary line between the SMV and SMA, and the MCA root dissected into its right branch [2].

### 2.4. Pathologic Evaluation

In the FLNM group, a laparoscopic NIR camera guided the harvesting of fluorescent D3 LNs. These nodes were labeled according to their anatomical association with the colic arteries, including the ICG-ICA, RCA, and MCA LNs. In the control group, the LNs around the colonic artery ligation sites were visually examined without fluorescence imaging. The D3 LNs were harvested on an auxiliary table and labeled as ICA, RCA, or MCA LNs based on their location. A pathologist evaluated the LNs from each labeled specimen in the pathology laboratory. They manually palpated and isolated the pericolic and intermediate LNs within the mesocolon. Harvested LNs were categorized into pericolic/intermediate and D3 areas. Pathologic evaluations of LNs were conducted to identify the metastatic nodes. The dissected LNs were fixed in formalin and processed following standard protocols. The nodes were then embedded in paraffin, sectioned at 4 µm thickness, and stained with hematoxylin and eosin.

### 2.5. Diagnostic Values of Preoperative CT Staging

The diagnostic values of preoperative CT staging were assessed using confusion matrix in both the FLNM and control groups by calculating the apparent prevalence, true prevalence, sensitivity, specificity, positive predictive value (PPV), negative predictive value (NPV), positive likelihood ratio (PLR), negative likelihood ratio (NLR), false positive and false negative proportions, and accuracy (Table 1). The PPV reflects the probability that a positive finding on preoperative CT corresponds to a true-positive outcome, indicating the presence of metastatic LNs. The PLR is an indicator of the likelihood of a positive test result such as a suspicious LN on CT in patients with metastatic disease compared to those without. A higher PLR indicated a more effective test. The false T+ proportion for true D− refers to the rate of false positives among true negatives, whereas the false T+ proportion for T+ refers to the rate of false positives among all positive test results. The formulae used for these calculations followed the methods described in a previous study [20]. CT staging was categorized as over-staging if it exceeded pathologic staging and under-staging if it was lower.

### 2.6. Statistical Analysis

Statistical analyses were performed to compare outcomes between the FLNM and conventional groups. Categorical variables were assessed using Pearson’s Chi-square and Fisher’s exact tests, while Student’s *t*-test was used to compare the mean values of harvested and metastatic LNs. Diagnostic values were analyzed using a confusion matrix and 95% confidence intervals (CIs) were computed and compared using forest plots. Multivariate analysis was performed with binary logistic regression model using a forward step wise analysis. The covariance input criterion was less than 0.1 and elimination criterion was less than 0.05. Analyses were conducted using R software (version 4.3.0; R Foundation for Statistical Computing, Vienna, Austria). Statistical significance was determined using a two-tailed *p*-value < 0.05.

## 3. Results

### 3.1. Patient’s Characteristics

The clinicopathologic characteristics were comparable between the FLNM and control groups (Table 2). No adverse events were associated with the endoscopic submucosal ICG injection for FLNM.

### 3.2. CT Staging and Pathologic Diagnosis

**Figure 3:**
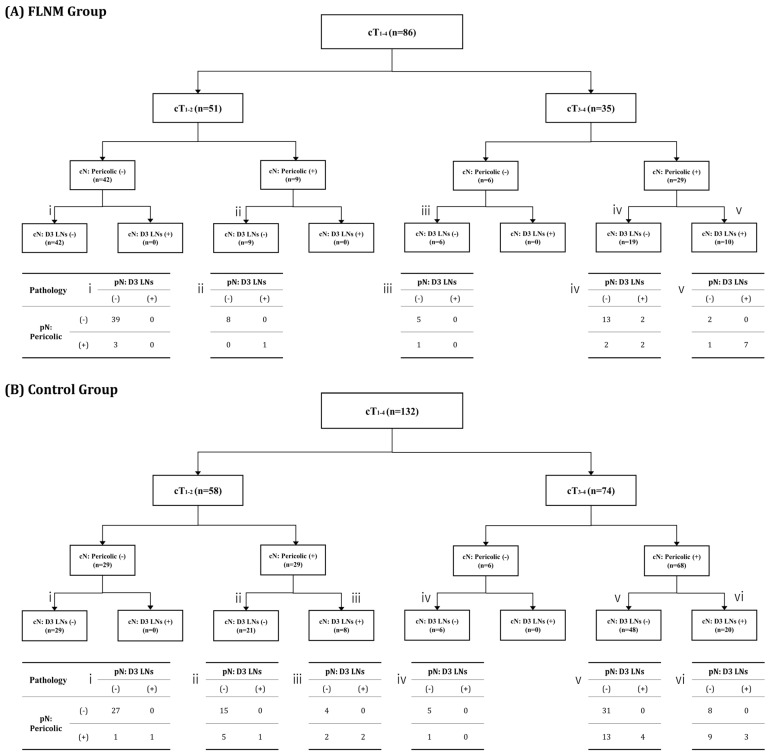
Flowchart of the diagnostic process for sequential classification of CT staging into cT and cN statuses. (**A**) FLNM group. (**B**) Control group. The mini-table (i–vi) presents the pathologic results of the LNs corresponding to the flowchart categories. cT and cN denote clinical statuses, while pN represents pathologic status.

The diagnostic process for the sequential classification of CT staging into cT and cN statuses is illustrated in a flow chart (Figure 3). For each staging classification, the pathologic results of the LNs are presented in a mini-table. In the cT_1-2_ status, cN staging indicated that pericolic LN metastasis (17.6% vs. 50.0%, *p* < 0.001) and D3 LN metastasis (0% vs. 13.8%, *p* = 0.005) were less frequently assessed in the FLNM group than in the control group. However, regarding pN staging, there was no statistically significant difference between the FLNM and control groups in terms of pericolic LN metastasis (7.8% vs. 13.8%, *p* = 0.322) or D3 LN metastasis (2.0% vs. 3.5%, *p* = 0.636).

In the cT_3-4_ status, the FLNM and control groups showed similar rates of pericolic LN metastasis (82.9% vs. 91.9%, *p* = 0.159) and D3 LN metastasis (28.6% vs. 27.0%, *p* = 0.866) according to cN staging. While there was no significant difference in pericolic LN metastasis (37.1% vs. 40.5%, *p* = 0.735) in pN staging between the two groups, D3 LN metastasis was significantly more frequent in the FLNM group (31.4% vs. 9.5%, *p* = 0.004).

The frequency of pathologic LN metastasis increased with a higher cT status (Table 3). LN metastasis confined to the pericolic region was observed in 17.4% of all cases, while continuous metastasis extending to D3 LNs occurred in 9.6%. Notably, skip metastasis, where the D3 LN metastasis occurred without pericolic LN involvement, was identified in 0.9% of all cases. D3 LN metastasis was observed only in those with cT_3-4_ status.

### 3.3. Diagnostic Value of CT Staging

The accuracy of CT staging in predicting LN metastasis was analyzed according to LN location (Table 4). For pericolic LNs, the accuracy of cN staging exceeded 92% in patients with early stage cancer (cT_1-2_N_0_). However, in patients with advanced colon cancer (cT_3-4_N_0_/cT_Any_N_1-2_) requiring D3 LND, the accuracy dropped to 40–42%, primarily due to over-staging in more than 56% of both groups. There was no significant difference in the cN staging accuracy of pericolic LNs between the FLNM and control groups.

The accuracy for the D3 LNs was more than 96% in patients with early cancer, with no significant difference between the FLNM and control groups. In contrast, in patients with advanced cancer, the accuracy was significantly different between the FLNM and control groups, at 81.8% and 72.8%, respectively. Over-staging was significantly lower in the FLNM group than in the control group (6.8% vs. 22.3%, respectively).

**Figure 4:**
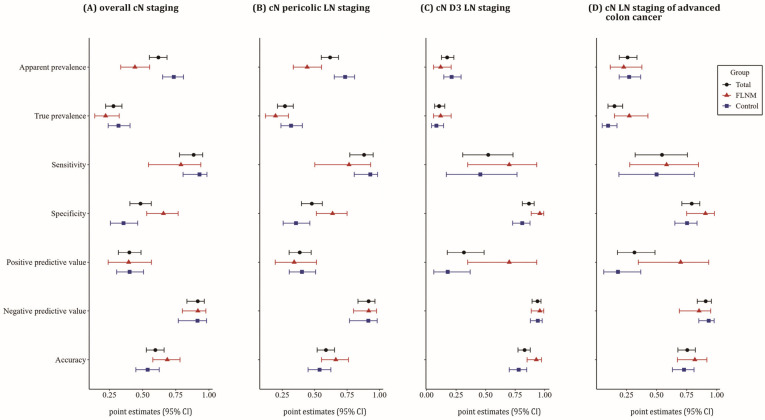
Forest plot of diagnostic values for cN staging, comparing the FLNM and control groups. Analyses include overall cN staging (**A**), pericolic LN staging (**B**), D3 LN staging (**C**), and cN staging in advanced colon cancer (cT_3-4_N_0_ or cT_ANY_N_1-2_) (**D**). The 95% confidence intervals (point estimates) are depicted with red line (triangle) for the FLNM group, blue line (square) for the control group, and black line (circle) for total cases. The apparent prevalence of cN positivity was significantly higher in the control group, driven by the significantly higher pericolic LN positivity. However, the true prevalence showed no statistical difference between the FLNM and control group. The diagnostic specificity of cN staging was improved in the FLNM group. Additionally, the positive predictive value and accuracy of D3 LN staging were enhanced in the FLNM group. In advanced colon cancer patients, the FLNM group tended to have higher true prevalence and positive predictive value.

When comparing the diagnostic values of cN assessment between the FLNM and the control group, the apparent prevalence of LN metastasis was higher in the control group, while the true prevalence was similar between both groups. This finding was consistent with the results observed for pericolic LNs. In D3 LNs, there was no difference in apparent prevalence, although the true prevalence tended to be higher in the FLNM group at advanced colon cancers. The sensitivity was comparable between both groups; however, the specificity was notably higher in the FLNM group for cN stage, pericolic LNs, and D3 LNs. The PPV and accuracy were similar between groups for pericolic LNs, but the FLNM group tended to have higher values for D3 LNs (Figure 4).

**Figure 5:**
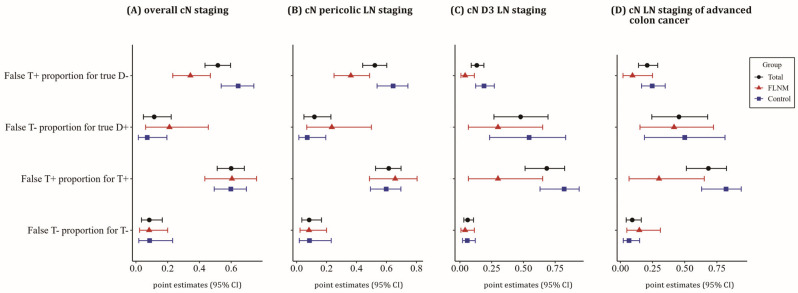
Forest plot of diagnostic values related to false-positive and negative rates. Analyses include overall cN staging (**A**), cN pericolic LN staging (**B**), cN D3 LN staging (**C**), and cN staging in advanced colon cancer (cT_3-4_N_0_ or cT_ANY_N_1-2_) (**D**). The 95% confidence intervals (point estimates) are depicted with red line (triangle) for the FLNM group, blue line (square) for the control group, and black line (circle) for total cases. The FLNM group exhibited a lower false-positive rate for true-negative cases (false T+ proportion for true D−), and a trend toward a lower false-positive rate in test-positive cases (false T+ proportion for T+) for D3 LNs. In advanced colon cancer, the FLNM group demonstrated a reduced false-positive rate for test-positive cases.

The FLNM group also showed a lower false-positive rate for true-negative cases (false T+ proportion for true D−) and a trend toward a lower false-positive rate in test-positive cases (false T+ proportion for T+) for D3 LNs (Figure 5).

The PLR for D3 LNs was significantly higher in the FLNM group compared to the control group (17.27 vs. 2.39, respectively). In early colorectal cancer (cT_1-2_N_0_), there were no significant differences in diagnostic values of cN staging between the both groups. However, in advanced stage (cT_3-4_N_0_ or cT_ANY_N_1-2_), the FLNM group demonstrated improved diagnostic parameters, including a higher true prevalence of LN metastasis, an increased PPV, and a reduced false-positive rate for test-positive cases (Supplement Tables S1–S5).

### 3.4. Univariate and Multivariate Analysis

The clinicopathologic factors associated with pathologic D3 LN metastasis are shown in Table 5. Multivariate analysis identified pN staging, vascular invasion, perineural invasion, cancer cell differentiation, and FLNM as independent factors associated with pathologic D3 LN metastasis.

## 4. Discussion

FLNM has emerged as a promising tool that offers a real-time visualization of lymphatic structures during surgery [21]. This method could enhance the surgeon’s ability to identify and excise D3 LNs that may harbor metastatic cells, especially in advanced colon cancer. In D3 LND, where LNs are located deep in the mesentery near critical vascular structures, the ability to visually distinguish these nodes in real-time is invaluable as it minimizes the damage to adjacent organs and helps reduce postoperative complications [22]. FLNM offers significant benefits in colorectal cancer surgery, including the improved detection of D3 LNs that may not be visible on conventional imaging during standard surgical exploration, which is essential for achieving thorough oncological resections and accurate staging [13,23,24]. Additionally, FLNM reduces the risk of missing metastatic D3 LNs and, consequently, lowers the risk of disease recurrence [25]. Furthermore, the real-time visualization of LNs provided by FLNM supports more precise surgical D3 LND [15,16]. These advantages suggest that FLNM can enhance the overall quality of colon cancer surgery, particularly in cases in which D3 LN metastasis is suspected based on preoperative CT findings.

Regarding the impact of FLNM on the diagnostic values of preoperative CT staging, the integration of FLNM into D3 LND has been shown to improve several key diagnostic metrics, including the true prevalence, PPV, PLR, and accuracy, while reducing false-positive rates. These metrics are critical for evaluating the effectiveness of preoperative CT for predicting LN metastasis and guiding surgical decisions.

This study found that the FLNM group demonstrated a higher PPV and PLR for D3 LN metastasis than in the control group, indicating an improved alignment between the CT findings and pathologic outcomes facilitated by FLNM. The improved PPV and PLR can be attributed to FLNM’s ability to enhance the precision of D3 LN identification and dissection, ensuring that positive findings on CT scans were more likely to represent true metastasis. FLNM also reduces false-positive rates for D3 LNs, which is crucial to avoid unnecessary treatments and surgeries, which can lead to increased patient morbidity and healthcare costs. We would like to emphasize the observation that false positives for D3 LNs were higher in the control group, potentially due to incomplete or inadequate D3 LND during surgery. In contrast, FLNM enables the completed removal of D3 LNs identified as metastatic during radiological staging, increasing the likelihood of confirming pathologic metastasis and improving diagnostic accuracy.

Furthermore, this study found that the accuracy of D3 LN staging was significantly higher in the FLNM group than in the control group. This trend suggests that FLNM contributes to more reliable preoperative D3 LN staging results, which are critical for effective surgical planning and improved patient outcomes.

The benefits of FLNM were more pronounced in advanced colon cancer cases (cT_3-4_N_0_ or cT_ANY_N_1-2_), where the likelihood of LN metastasis is higher and preoperative CT imaging may struggle to accurately distinguish between benign and malignant nodes due to the complex lymphatic involvement. In such cases, FLNM provides greater precision by enabling targeted dissection of D3 LNs, thereby enhancing diagnostic accuracy. Conversely, in early colon cancer (cT_1-2_N_0_), where the likelihood of LN metastasis is lower, preoperative CT may provide a relatively accurate LN assessment, resulting in less pronounced benefits from FLNM. Alternatively, FLNM may be useful for sentinel LN identification in early-stage colon cancer [26].

The observed improvements in diagnostic accuracy with FLNM can be attributed to several underlying mechanisms. FLNM enhances the real-time visualization of LNs during surgery, enabling more precise D3 LND and the removal of nodes that are most likely to be metastatic [27]. This enhanced accuracy reduces the likelihood of radiological over-staging. Additionally, FLNM improves the correlation between preoperative imaging and pathologic findings, increasing the probability that LNs identified as suspicious on CT will be confirmed as metastatic on pathologic examination, thereby enhancing the overall diagnostic accuracy of preoperative CT staging.

Previous studies support that FLNM improves surgical quality in gastrointestinal cancer surgery. FLNM can improve the harvested LN counts by increasing the precision of LND for colorectal cancer surgery [15,16] FLNM has been shown to improve surgical outcomes by ensuring the complete resection of the involved LNs for gastric cancer study [28]. In this study, FLNM was identified as an independent factor associated with D3 LN metastasis, suggesting that its use of FLNM during surgery can enable more precise D3 LND, thereby enhancing the removal of metastatic nodes. Accurate LN staging is critical for determining appropriate postoperative treatment, including the need for adjuvant chemotherapy [29,30]. By improving the accuracy of preoperative CT imaging, FLNM ensures that patients receive the most appropriate treatment for their disease, leading to improved overall outcomes.

In this study, D3 LND was performed regardless of CT stage, comparing the presence of nodal metastasis with CT prediction results. Consequently, there was no difference in the surgical protocol between patients undergoing FLNM and those in the control group. Notably, the harvested D3 LN count and the detection rate of metastatic D3 LNs were significantly higher in the FLNM group, suggesting that FLNM enhances visualization of the D3 LN region and may necessitate additional radical dissection in some patients compared to conventional D3 LND. Fluorescent LNs around the middle colic artery often extended toward the pancreas, allowing for LND even in deep areas obscured by mesenteric fat tissue. Therefore, FLNM may facilitate complete D3 LND in advanced colon cancer with potential D3 LN metastasis. Conversely, no patients with CT staging of cT_1-2_N_0_M_0_ had D3 LN metastasis in this study. Thus, future studies could consider fluorescence-guided D3 LN excision based on FLNM instead of radical D3 LND in patients with early-stage colon cancer (cT_1-2_N_0_M_0_). Radical D3 LND is associated with longer operative times and increased risks, including vascular injury, massive bleeding, conversion to open surgery, and postoperative chyle ascites, making it suitable only for more experienced surgeons. In early colon cancer, FLNM-based fluorescence D3 LN excision can selectively remove major D3 LNs linked to the primary tumor, offering a simpler and safer approach. Additionally, pathologic examination can confirm the presence of D3 LN metastasis, enabling a customized surgical strategy based on CT staging.

This study had several strengths and limitations. The study’s innovative approach, which incorporates FLNM into the surgical process, offers a novel method for improving the accuracy of LND and enhancing the diagnostic values of preoperative CT staging. A comprehensive analysis of various diagnostic metrics, including PPV, PLR, and accuracy, provided a robust evaluation of the impact of FLNM on surgical and diagnostic outcomes. However, potential weaknesses include the possibility of a limited sample size, which could affect the generalizability of the findings, and a single-center design, which could restrict the applicability of the results across different clinical settings. Additionally, there is a potential for selection bias due to variations in surgical techniques and patient characteristics arising from differences in the timing of surgery between the FLNM and control group. This study compared patients undergoing FLNM from 2018 to 2023 with those who did not. FLNM was primarily performed starting in 2020, following the implementation of a standardized protocol based on a preceding standardization protocol. The control group mainly includes surgeries from 2018 to 2019, while the FLNM group consists of surgeries from 2020 to 2023. Patients who did not undergo FLNM for various reasons after 2020 were included in the control group. Differences in surgical timing may reflect variations in surgeon experience. In this study, randomization was not performed to divide patients into two groups, as FLNM was attempted in all feasible cases. While the most scientific approach to verify the effectiveness of a new surgical or examination method is a prospective randomized study, this initial case–control study was conducted to introduce FLNM and explore its potential utility. A multicenter prospective randomized study is currently underway to validate the effectiveness of FLNM observed in this study. The inherent limitations of retrospective CT staging remain, and since the radiologic staging was conducted by a single radiologist, individual variations in assessment may impact the validity of the radiologic diagnosis [12]. In this study, CT evaluations for colon cancer staging were performed by a single radiologist specializing in colon cancer at our institution. We recognized the potential bias due to differences in the radiologist’s experience, particularly given the 6-year enrollment period, which represents a structural limitation of the study. To address this, the radiologist re-evaluated the CT scans of all enrolled patients, with blinding of patient information, pathologic results, and other relevant data. As a result, discrepancies were noted between the initial CT staging at diagnosis and the re-evaluated CT staging in some patients. However, this re-evaluation was conducted to minimize bias associated with the radiologist’s evaluation period after establishment of clear criteria for assessing metastatic LNs, which could be subjectively interpreted, and the re-evaluated CT staging was used in the analysis. Since the initial evaluation and re-evaluation were both performed by the same radiologist, interobserver reliability—which is typically assessed when multiple radiologists are involved—was not analyzed in this study. This could affect the validity of the conclusions and should be addressed in future research. Additionally, we did not describe the long-term oncologic outcomes in this study. Given the inclusion of various stages, we compared survival rates between the FLNM and control groups at each stage. No significant differences were found in stages I and II; however, the FLNM group showed a 10% improvement in disease-free survival in stage III, which did not reach statistical significance, likely due to the small sample size. Since this study was not designed to evaluate FLNM’s effect on survival rates, these comparisons have limited statistical value. A large-scale study is planned to investigate the oncologic benefits associated with improved surgical quality from FLNM. Further research, particularly multicenter studies would be beneficial to confirm these findings and establish the role of FLNM in improving the diagnostic accuracy of preoperative CT in colon cancer surgery.

The primary limitation of FLNM using ICG is its tendency to produce non-specific fluorescence expression in cancerous tissues. During our study, we observed that heterogenous fluorescence patterns may arise when metastatic LNs partially replace normal LN tissue. Consequently, artificial intelligence-based analysis of heterogeneous fluorescence patterns might enable the prediction of metastatic LNs, similar to the assessment of LN metastasis using parenchymal heterogeneity in CT imaging. Additionally, the development of novel cancer-specific fluorescence imaging techniques, utilizing reagents that conjugate fluorescent dyes with cancer-specific antibodies, is anticipated to be introduced into clinical practice in the near future [31,32].

## 5. Conclusions

The use of FLNM for D3 LND enhances the diagnostic accuracy of cN staging in right-sided colon cancer by improving surgical precision. This advancement could lead to more accurate staging, better treatment planning, and improved patient outcomes, especially for advanced colon cancer.

## Figures and Tables

**Table 1:** Confusion matrix.

	Reference (True)	
Predicted	Event	No Event
Event	A	B
No Event	C	D
(1)	Apparent prevalence (or detection prevalence) = (A + B)/(A + B + C + D)	
(2)	True prevalence = (A + C)/(A + B + C + D)	
(3)	Sensitivity = A/(A + C)	
(4)	Specificity = D/(B + D)	
(5)	Positive predictive value = A/(A + B)	
(6)	Negative predictive value = D/(C + D)	
(7)	Positive likelihood ratio = Sensitivity/1 − Specificity	
(8)	Negative likelihood ratio = 1 − Sensitivity/Specificity	
(9)	False T+ proportion for true D− = B/(B + D)	
(10)	False T− proportion for true D+ = C/(A + C)	
(11)	False T+ proportion T+ = B/(A + B)	
(12)	False T− proportion T− = C/(C + D)	
(13)	Correctly classified proportion (Accuracy) = (A + D)/(A + B + C + D)	

**Table 2:** Patient’s characteristics (*n* = 218).

Clinical Variables		FLNM (*n* = 86)	Control (*n* = 132)	*p*-Value
		*n* (%)	*n* (%)	
Age, yr	mean ± SD	67.7 ± 11.7	67.6 ± 10.5	0.974
Sex	male	45 (52.3)	62 (47.0)	0.439
	female	41 (47.7)	70 (53.0)	
BMI (kg/m^2^)	mean ± SD	23.7 ± 3.1	23.9 ± 2.8	0.602
Cancer location	cecum	10 (11.6)	20 (15.2)	0.538
	ascending colon	51 (59.3)	80 (60.6)	
	hepatic flexure	19 (22.1)	20 (15.2)	
	proximal transverse colon	6 (7.0)	12 (9.1)	
cT status	cT_1-2_	51 (59.3)	58 (43.9)	0.027
	cT_3-4_	35 (40.7)	74 (56.1)	
cN status	cN_0_	48 (55.8)	35 (26.5)	0.000
	cN_1-2_	38 (44.2)	97 (73.5)	
pT status	pT_1-2_	38 (44.2)	39 (29.5)	0.027
	pT_3-4_	48 (55.8)	93 (70.5)	
pN status	pN_0_	67 (77.9)	90 (68.2)	0.118
	pN_1-2_	19 (22.1)	42 (31.8)	
Pathologic stage	I	37 (43.0)	34 (25.8)	0.028
	II	29 (33.7)	55 (41.7)	
	III	20 (23.3)	43 (32.6)	
Tumor size (cm)	mean ± SD	3.8 ± 2.2	4.4 ± 2.4	0.078
Differentiation	well	42 (48.8)	27 (20.5)	<0.001
	moderate	42 (48.8)	97 (73.5)	
	poorly	2 (2.3)	8 (6.1)	
Cancer obstruction	positive	16 (18.6)	28 (21.2)	0.639
Lymphatic invasion	positive	14 (16.3)	26 (19.7)	0.524
Vascular invasion	positive	4 (4.7)	14 (10.6)	0.118
Perineural invasion	positive	17 (19.8)	26 (19.7)	0.990

FLNM; fluorescence lymph node mapping, SD; standard deviation, BMI; body mass index.

**Table 3:** The frequency of pathologic lymph node (LN) metastasis according to cT status.

	cT_1_	cT_2_	cT_3_	cT_4_	Total	*p*-Value	
		*n* (%)					
**FLNM (*n* = 86)**							
LN (−)	24 (100.0)	23 (85.2)	10 (62.5)	10 (52.6)	67 (77.9)	0.001	
LN (+)							
Pelicolic LN	0 (0.0)	3 (11.1)	1 (6.3)	3 (15.8)	7 (8.1)		
D3 LN	0 (0.0)	0 (0.0)	1 (6.3)	1 (5.3)	2 (2.3)		
Pelicolic + D3 LN	0 (0.0)	1 (3.7)	4 (25.0)	5 (26.3)	10 (11.6)		
**Control (*n* = 132)**							
LN (−)	16 (94.1)	30 (73.2)	22 (68.8)	22 (52.4)	90 (68.2)	0.081	
LN (+)							
Pelicolic LN	1 (5.9)	7 (17.1)	8 (25.0)	15 (35.7)	31 (23.5)		
D3 LN	0 (0.0)	0 (0.0)	0 (0.0)	0 (0.0)	0 (0.0)		
Pelicolic + D3 LN	0 (0.0)	4 (9.8)	2 (6.3)	5 (11.9)	11 (8.3)		
**Total (*n* = 218)**							
LN (−)	40 (97.6)	53 (77.9)	32 (66.7)	32 (52.5)	157 (92.0)	<0.001	
LN (+)							
Pelicolic LN	1 (2.4)	10 (14.7)	9 (18.8)	18 (29.5)	38 (17.4)		
D3 LN	0 (0.0)	0 (0.0)	1 (2.1)	1 (1.6)	2 (0.9)		
Pelicolic + D3 LN	0 (0.0)	5 (7.4)	6 (12.5)	10 (16.4)	21 (9.6)		

LN (+) is categorized as follows: cases where metastasis is confirmed solely in the pericolic LN or D3, and cases where metastasis is confirmed in both the pericolic and D3 LN.

**Table 4:** Accuracy, under-staging, and over-staging of cN according to lymph node (LN) locations.

**cN: pericolic LN**					
**cT_1-2_N_0_**		**FLNM, *n* (%)**	**Control, *n* (%)**	**Total, *n* (%)**	***p* value**
	Accuracy	39 (92.9)	27 (93.1)	66 (93.6)	1.000
	Under-staging	3 (7.1)	2 (6.9)	5 (7.0)	
	Total	42 (100)	29 (100)	71 (100)	
**cT_3-4_N_0_/cT_Any_N_1-2_**		**FLNM, *n* (%)**	**Control, *n* (%)**	**Total, *n* (%)**	***p* value**
	Accuracy	18 (40.9)	44 (42.7)	62 (42.2)	0.797
	Over-staging	25 (56.8)	58 (56.3)	83 (56.5)	
	Under-staging	1 (2.3)	1 (1.0)	2 (1.4)	
	Total	44 (100)	103 (100)	147 (100)	
**cN: D3 LN**					
**cT_1-2_N_0_**		**FLNM, *n* (%)**	**Control, *n* (%)**	**Total, *n* (%)**	***p* value**
	Accuracy	42 (100)	28 (96.6)	70 (98.6)	0.408
	Under-staging	0 (0)	1 (3.4)	1 (1.4)	
	Total	42 (100)	29 (100)	71 (100)	
**cT_3-4_N_0_/cT_Any_N_1-2_**		**FLNM, *n* (%)**	**Control, *n* (%)**	**Total, *n* (%)**	***p* value**
	Accuracy	36 (81.8)	75 (72.8)	111 (75.5)	0.031
	Over-staging	3 (6.8)	23 (22.3)	26 (17.7)	
	Under-staging	5 (11.4)	5 (4.9)	10 (6.8)	
	Total	44 (100)	103 (100)	147 (100)	

**Table 5:** Univariate and multivariate analysis for clinicopathologic factors associated with pathologic D3 LN metastasis.

Characteristics	Univariate Analysis			Multivariate Analysis			
	Pathologic D3 LN Metastasis		*p*-Value	B	Exp (B)	95% CI	*p*-Value
	Negative	Positive					
Cancer obstruction	34 (17.4)	10 (43.5)	0.003				
cT_3-4_	91 (46.7)	18 (78.3)	0.004				
cN_1-2_	113 (57.9)	22 (95.7)	<0.001				
cN pericolic LN (+)	113 (57.9)	22 (95.7)	<0.001				
cN D3 LN (+)	26 (13.3)	12 (52.2)	<0.001	2.478	11.917	3.507–40.492	<0.001
Lymphatic invasion	28 (14.4)	12 (52.2)	<0.001				
Vascular invasion	11 (5.6)	7 (30.4)	<0.001	2.399	11.017	2.712–44.765	<0.001
Perineural invasion	30 (15.4)	13 (56.5)	<0.001	1.711	5.537	1.884–16.269	0.002
Differentiation (moderate to poor)	128 (65.6)	21 (91.3)	0.012	2.108	8.228	1.635–49.688	0.021
FLNM	74 (37.9)	12 (52.2)	0.187	1.950	7.028	2.081–23.737	0.002

LN; lymph node, FLNM; fluorescence lymph node mapping, B; beta coefficient, Exp (B); exponentiated regression coefficient as odds ratio, CI; confidence interval.

## Data Availability

The data presented in this study are available on request from the corresponding author. The data are not publicly available due to ethical and privacy reasons.

## Supplementary Materials

The following supporting information can be downloaded at: https://www.mdpi.com/article/10.3390/cancers16203496/s1, Table S1: Confusion Matrix and Statistics for overall cN staging; Table S2: Confusion Matrix and Statistics for cN pericolic LN staging; Table S3: Confusion Matrix and Statistics for cN D3 LN staging; Table S4: Confusion Matrix and Statistics for cN LN staging of early colon cancer; Table S5: Confusion Matrix and Statistics for cN LN staging of advanced colon cancer.
