# Supplementary material for: Effect of Fluorescence Lymph Node Mapping on Improving Diagnostic Values of CT D3 Lymph Node Staging for Right-Sided Colon Cancer
Source: Cancers (Basel). 2024 Oct 16;16(20):3496. doi: 10.3390/cancers16203496 (PMC11505676; doi:10.3390/cancers16203496)
Supplement: Supplementary file 1 [file cancers-16-03496-s001.zip › cancers-3217014-supplementary.pdf]

Supplement Table S1. Confusion Matrix and Statistics for overall cN staging

Total LN	FLNM (*n* = 86)	Control (*n* = 132)	Total (*n* = 218)
	Point estimates (95% CI)		
Apparent prevalence	0.442 (0.335, 0.553)	0.735 (0.651, 0.808)	0.619 (0.551, 0.684)
True prevalence	0.221 (0.139, 0.323)	0.318 (0.240, 0.405)	0.280 (0.221, 0.344)
Sensitivity	0.789 (0.544, 0.939)	0.929 (0.805, 0.985)	0.885 (0.778, 0.953)
Specificity	0.657 (0.531, 0.768)	0.356 (0.257, 0.463)	0.484 (0.404, 0.565)
Positive predictive value	0.395 (0.240, 0.566)	0.402 (0.304, 0.507)	0.400 (0.317, 0.488)
Negative predictive value	0.917 (0.800, 0.977)	0.914 (0.769, 0.982)	0.916 (0.834, 0.965)
Positive likelihood ratio	2.300 (1.535, 3.446)	1.441 (1.210, 1.716)	1.716 (1.438, 2.047)
Negative likelihood ratio	0.321 (0.132, 0.779)	0.201 (0.065, 0.619)	0.237 (0.116, 0.485)
False T+ proportion for true D-	0.343 (0.232, 0.469)	0.644 (0.537, 0.743)	0.516 (0.435, 0.596)
False T- proportion for true D+	0.211 (0.061, 0.456)	0.071 (0.015, 0.195)	0.115 (0.047, 0.222)
False T+ proportion for T+	0.605 (0.434, 0.760)	0.598 (0.493, 0.696)	0.600 (0.512, 0.683)
False T- proportion for T-	0.083 (0.023, 0.200)	0.086 (0.018, 0.231)	0.084 (0.035, 0.166)
Correctly classified proportion	0.686 (0.577, 0.782)	0.538 (0.449, 0.625)	0.596 (0.528, 0.662)

FLNM; fluorescence lymph node mapping, LN; lymph node, CI; confidence interval

Supplement Table S2. Confusion Matrix and Statistics for cN pericolic LN staging

Pericolic LN	FLNM (*n* = 86)	Control (*n* = 132)	Total (*n* = 218)
	Point estimates (95% CI)		
Apparent prevalence	0.442 (0.335, 0.553)	0.735 (0.651, 0.808)	0.619 (0.551, 0.684)
True prevalence	0.198 (0.120, 0.298)	0.318 (0.240, 0.405)	0.271 (0.213, 0.335)
Sensitivity	0.765 (0.501, 0.932)	0.929 (0.805, 0.985)	0.881 (0.771, 0.951)
Specificity	0.638 (0.513, 0.750)	0.356 (0.257, 0.463)	0.478 (0.398, 0.559)
Positive predictive value	0.342 (0.196, 0.514)	0.402 (0.304, 0.507)	0.385 (0.303, 0.473)
Negative predictive value	0.917 (0.800, 0.977)	0.914 (0.769, 0.982)	0.916 (0.834, 0.965)
Positive likelihood ratio	2.111 (1.402, 3.178)	1.441 (1.210, 1.716)	1.688 (1.416, 2.013)
Negative likelihood ratio	0.369 (0.154, 0.885)	0.201 (0.065, 0.619)	0.248 (0.122, 0.507)
False T+ proportion for true D-	0.362 (0.250, 0.487)	0.644 (0.537, 0.743)	0.522 (0.441, 0.602)
False T- proportion for true D+	0.235 (0.068, 0.499)	0.071 (0.015, 0.195)	0.119 (0.049, 0.229)
False T+ proportion for T+	0.658 (0.486, 0.804)	0.598 (0.493, 0.696)	0.615 (0.527, 0.697)
False T- proportion for T-	0.083 (0.023, 0.200)	0.086 (0.018, 0.231)	0.084 (0.035, 0.166)
Correctly classified proportion	0.663 (0.553, 0.761)	0.538 (0.449, 0.625)	0.587 (0.519, 0.653)

FLNM; fluorescence lymph node mapping, LN; lymph node, CI; confidence interval

Supplement Table S3. Confusion Matrix and Statistics for cN D3 LN staging

D3 LN	FLNM (*n* = 86)	Control (*n* = 132)	Total (*n* = 218)
	Point estimates (95% CI)		
Apparent prevalence	0.119 (0.059, 0.208)	0.212 (0.146, 0.292)	0.174 (0.126, 0.231)
True prevalence	0.119 (0.059, 0.208)	0.083 (0.042, 0.144)	0.106 (0.068, 0.154)
Sensitivity	0.700 (0.348, 0.933)	0.455 (0.167, 0.766)	0.522 (0.306, 0.732)
Specificity	0.959 (0.886, 0.992)	0.810 (0.729, 0.876)	0.867 (0.811, 0.911)
Positive predictive value	0.700 (0.348, 0.933)	0.179 (0.061, 0.369)	0.316 (0.175, 0.487)
Negative predictive value	0.959 (0.886, 0.992)	0.942 (0.879, 0.979)	0.939 (0.893, 0.969)
Positive likelihood ratio	17.267 (5.304, 56.211)	2.391 (1.136, 5.035)	3.913 (2.303, 6.650)
Negative likelihood ratio	0.313 (0.121, 0.807)	0.673 (0.390, 1.163)	0.552 (0.359, 0.849)
False T+ proportion for true D-	0.041 (0.008, 0.114)	0.190 (0.124, 0.271)	0.133 (0.089, 0.189)
False T- proportion for true D+	0.300 (0.067, 0.652)	0.545 (0.234, 0.833)	0.478 (0.268, 0.694)
False T+ proportion for T+	0.300 (0.067, 0.652)	0.821 (0.631, 0.939)	0.684 (0.513, 0.825)
False T- proportion for T-	0.041 (0.008, 0.114)	0.058 (0.021, 0.121)	0.061 (0.031, 0.107)
Correctly classified proportion	0.929 (0.851, 0.973)	0.780 (0.700, 0.848)	0.830 (0.774, 0.878)

FLNM; fluorescence lymph node mapping, LN; lymph node, CI; confidence interval

Supplement Table S4. Confusion Matrix and Statistics for cN LN staging of early colon cancer

Total LN	FLNM (*n* = 42)	Control (*n* = 29)	Total (*n* = 71)
	Point estimates (95% CI)		
Apparent prevalence	0.000 (0.000, 0.084)	0.000 (0.000, 0.119)	0.000 (0.000, 0.051)
True prevalence	0.000 (0.000, 0.084)	0.034 (0.001, 0.178)	0.014 (0.000, 0.076)
Sensitivity	NaN (0.000, 1.000)	0.000 (0.000, 0.975)	0.000 (0.000, 0.975)
Specificity	1.000 (0.916, 1.000)	1.000 (0.877, 1.000)	1.000 (0.949, 1.000)
Positive predictive value	NaN (0.000, 1.000)	NaN (0.000, 1.000)	NaN (0.000, 1.000)
Negative predictive value	1.000 (0.916, 1.000)	0.966 (0.822, 0.999)	0.986 (0.924, 1.000)
Positive likelihood ratio	NaN (NaN, NaN)	NaN (NaN, NaN)	NaN (NaN, NaN)
Negative likelihood ratio	NaN (NaN, NaN)	1.000 (1.000, 1.000)	1.000 (1.000, 1.000)
False T+ proportion for true D-	0.000 (0.000, 0.084)	0.000 (0.000, 0.123)	0.000 (0.000, 0.051)
False T- proportion for true D+	NaN (0.000, 1.000)	1.000 (0.025, 1.000)	1.000 (0.025, 1.000)
False T+ proportion for T+	NaN (0.000, 1.000)	NaN (0.000, 1.000)	NaN (0.000, 1.000)
False T- proportion for T-	0.000 (0.000, 0.084)	0.034 (0.001, 0.178)	0.014 (0.000, 0.076)
Correctly classified proportion	1.000 (0.916, 1.000)	0.966 (0.822, 0.999)	0.986 (0.924, 1.000)

FLNM; fluorescence lymph node mapping, LN; lymph node, CI; confidence interval, NaN; Not a Number

Supplement Table S5. Confusion Matrix and Statistics for cN LN staging of advanced colon cancer

Total LN	FLNM (*n* = 44)	Control (*n* = 103)	Total (*n* = 147)
	Point estimates (95% CI)		
Apparent prevalence	0.227 (0.115, 0.378)	0.272 (0.189, 0.368)	0.259 (0.190, 0.337)
True prevalence	0.273 (0.150, 0.428)	0.097 (0.048, 0.171)	0.150 (0.096, 0.218)
Sensitivity	0.583 (0.277, 0.848)	0.500 (0.187, 0.813)	0.545 (0.322, 0.756)
Specificity	0.906 (0.750, 0.980)	0.753 (0.652, 0.836)	0.792 (0.710, 0.859)
Positive predictive value	0.700 (0.348, 0.933)	0.179 (0.061, 0.369)	0.316 (0.175, 0.487)
Negative predictive value	0.853 (0.689, 0.950)	0.933 (0.851, 0.978)	0.908 (0.838, 0.955)
Positive likelihood ratio	6.222 (1.915, 20.221)	2.022 (0.990, 4.129)	2.622 (1.571, 4.377)
Negative likelihood ratio	0.460 (0.233, 0.906)	0.664 (0.354, 1.248)	0.574 (0.360, 0.915)
False T+ proportion for true D-	0.094 (0.020, 0.250)	0.247 (0.164, 0.348)	0.208 (0.141, 0.290)
False T- proportion for true D+	0.417 (0.152, 0.723)	0.500 (0.187, 0.813)	0.455 (0.244, 0.678)
False T+ proportion for T+	0.300 (0.067, 0.652)	0.821 (0.631, 0.939)	0.684 (0.513, 0.825)
False T- proportion for T-	0.147 (0.050, 0.311)	0.067 (0.022, 0.149)	0.092 (0.045, 0.162)
Correctly classified proportion	0.818 (0.673, 0.918)	0.728 (0.632, 0.811)	0.755 (0.677, 0.822)

FLNM; fluorescence lymph node mapping, LN; lymph node, CI; confidence interval
